# Polymer Translocation through Nanometer Pores

**DOI:** 10.3390/polym14061166

**Published:** 2022-03-15

**Authors:** Maria-Alexandra Paun, Vladimir-Alexandru Paun, Viorel-Puiu Paun

**Affiliations:** 1 School of Engineering, Swiss Federal Institute of Technology (EPFL), 1015 Lausanne, Switzerland; maria_paun2003@yahoo.com; 2 Division Radio Monitoring and Equipment, Section Market Access and Conformity, Federal Office of Communications OFCOM, Federal Department of the Environment, Transport, Energy and Communications DETEC, Rue de l’Avenir 44, 2501 Bienne, Switzerland; 3 Five Rescue Research Laboratory, 75004 Paris, France; vladimir.alexandru.paun@ieee.org; 4 Department of Physics, Faculty of Applied Sciences, University Politehnica of Bucharest, 060042 Bucharest, Romania; 5 Academy of Romanian Scientists, 050094 Bucharest, Romania

**Keywords:** polymer translocation, polymer flux, nanometer pores

## Abstract

In this paper the loaded polymer transport and its escape via a nanometer size aperture, virtually by nanomembrane, the polymer being moved by an exterior electrostatic field, has been studied. Assuming a linear dependency of the friction coefficient on the number of segments *m* and a parabolic behavior for the open-free (Gibbs) energy, in attendance of a present electrical potential across nanopore, an explicit flux formula for the polymers passed over a dimensional restricted pore, was derived. In addition, the linear polymers transport through a nanometer-sized pore under the action of a constant force is presented. The important mechanical effects of superimposed steady force and the monomers number of macromolecule chain on the polymer translocation process by nanomembranes, in a 2D diffusion model, have been demonstrated. The escape time by a three-dimensional graph as a function of the electric field intensity and monomers number of polymer was represented.

## 1. Introduction

The polymer translocation modeling (for example, the various macromolecules, and even nucleic acids and protein molecules) through narrow calibrated pores, of nanometer size (so-called “nanopores”), along the classical membranes and the polymer flux computation are difficult tasks [1,2,3,4,5,6]. In addition, the realistic treatment of this subject should take into account the complex nature of polymer-pore interaction.

A correct approach to this problem, for situations considered perfect, will be presented in this paper. The case of an ideal polymer in a bidimensional space, separated by a thin wall membrane, in the presence of a free energy barrier, has been considered.

Polymer chains interact to form a bulk polymer. The configuration of this interaction can be either amorphous or crystalline. The amorphous (non-crystalline) configuration refers to a random arrangement of neighbouring polymer chains. For interest, all polymers are amorphous at a sufficiently high temperature, or when in solution. Regular arrangement, where repeat units within the polymer chain form a crystalline structure, are also possible. This crystallization can occur both within and across neighbouring polymer chains to imbue some degree of crystallization to the polymer.

Polymer chain structures range from those with a paucity of internal conformational states, such as the extreme of rodlike chains formed by para-catenated aromatic chains or helix structures stabilized by secondary intramolecular bonding, to chains with an abundance of conformational states due to rotational isomerism about valence bonds along the chain backbone. This review is mainly concerned with the first category, the rodlike chain polymers and even a linear configuration, after all. Under certain conditions, interactions (contacts) among chain elements distantly connected along the chain contour can be neglected in describing the conformational states of chains [7,8,9].

In the paper, we considered a polymer with an ideal crystalline configuration. The conceptual chain without interactions is called an “unperturbed” chain. An immediate consequence of this property is that the mean-square end-to end distance of the chain scales with the number of repeat units *N* in the chain. In practice, in many cases, the averaged chain conformation obtaining in a dilute solution at a certain temperature called the theta temperature (or Flory temperature) *Θ* closely approximates that of the unperturbed chain.

**Figure 1:**
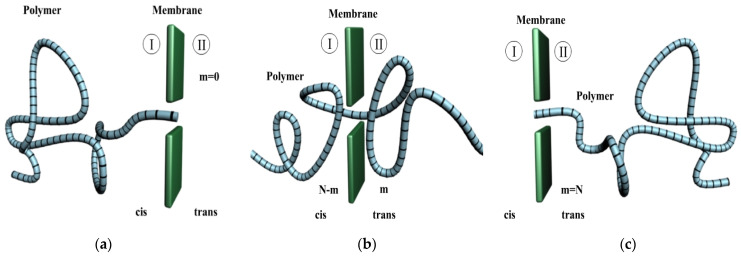
Polymer in transition through nanometer pore: (**a**) The charged polymer is found in the *cis* zone (region I), (**b**) The polymer has *m* monomers in the trans area and *N-m* monomers in the cis area, (**c**) The charged polymer is entirely found in the *trans* zone (region II).

The modeled system used throughout this work is based on Figure 1a–c. The ideal nanomembrane is entirely parallel to the *y*-*z* map-plan, and the center of the orifice is dimensionally a “point” on the *x*-axis. The pore is symmetric. The region I (*cis*) is found in the left side of the membrane, and in the right of the membrane we have the region II (*trans*).

The entire potential drop is assumed to be across the pore extension. Initially, the charged polymer is in the *cis* zone (Figure 1a), then passes through the pore (Figure 1b), and is finally contained in the *trans* zone (Figure 1c). The problem being physically symmetric, with respect to the membrane transversal plane, the electrical potential difference of the pores is considered reversible (change the sign), if the macromolecular chain is inserted on the trans side. In other words, the polymer transport process via a thin-walled membrane channel from an area with several possible configurations, into another area with a number of permitted configurations which acutely decrease, needs a special physical approach. Accordingly, the polymeric suite is displaced beyond an entropic type of border. In general, there are additional enthalpic contributions arising from the potential interaction among the various monomers constituting the polymer and the channel [4]. Therefore, the barrier encountered by the polymer is a free energy barrier.

Taking into account the speed developed in the displacement process, the translocation time, respectively the time in which the polymer passes with its full length from the cis zone to the trans zone, also called the escape time, expressed in accordance with the dynamic principles recognized in the model considered, has been calculated.

Much more, the same theoretical model leads to the establishment of the polymer flow through the pore, considered as a major objective of the research in our paper.

A general expression determination for the polymer flow through a small hole, located in a partition wall (seen as a porous nanomembrane), will be one of the successes of this present study.

In this article, we have three important chapters, followed by the bibliographic references. The first chapter is a consistent introduction, which presents the posed problem and the notions used in the other chapters. Chapter 2 is the chapter that develops the theory used in the paper, having two practically independent subsections. The first of these refers to the charged polymer translocation process through narrow protein pores, with a presentation in detail of a one-dimensional diffusion model for describing polymer motion through a nanopore, respectively the flux of polymers calculation. In the second subsection, the linear polymers transport through a nanometer sized pore under the action of a constant force is presented. The model proposed, which aims to accurately describe the polymer transport trough nanometric sized pores, relies on a drift-diffusion process and its associated equations. The last chapter is one of conclusions, which brings this study to a close.

## 2. Theoretical Model

### 2.1. Charged Polymer Translocation Process

In the study of the charged polymer translocation process through narrow protein pores, the realization of two research objectives (more important) are expected:

1. computation of time-evolution of translocation *τ,*
2. obtaining of the polymer flux through the pore *J*,

from which we are particularly interested in the latter.

These are the reasons that constrain us to examine both the macromolecule dynamic attitude in front of the nanopore and the screwing process in the pore. As we know, the two process coupling is essential. This is very important in understanding *J*, from the phenomenological point of view. In this sense, a detailed method to compute the flux, *J*, of the charged flexible polymers, in the company of the external electrostatic potential, through a narrow nanopore, is presented.

In our quantitative model of the polymer transport process, there are three key components:

(I) the free energy barrier estimation,
(II) the polymer evolution equations,
(III) the flux probability of translocation across the barrier.

#### 2.1.1. Free Energy

At the center of the wall there is a small pore that is wide enough for the polymer to thread through to the other side. The associated free energy, *F*(*m*), using polymer partition function *Z*, is

(1) $$F(m)=-k_{B}T\ln Z$$

or, under a chemical potential gradient [1],

(2) $$F(m)=-k_{B}T\ln Z+m\Delta \mu .$$

Here, in Equation (2), *Δμ* = *μ*_1_ − *μ*_2_ is the chemical potential difference per segment between inside and outside of the cell.

The partition sum *Z_m_*, in the case of macromolecule suite with *m* pieces (monomers) located in the cis area, conform to [10,11], will be

(3) $$Z_{m}{∼m}^{\gamma -1}$$

where γ=0.5 for Gaussian chains, γ≈0.69 for self-avoiding chains and γ=1 for rod-like chains.

For the *m* monomers in region II and *N*−*m* monomers of the polymer in region I, the associated free energy is

(4) $$\beta F(m)=\left(1-\gamma_{1}\right)\ln\left(N-m\right)+\left(1-\gamma_{2}\right)\ln\left(m\right)+m\beta \Delta \mu$$

where the constant terms are ignored and $\gamma_{1}$, respectively $\gamma_{2}$ are the values of γ in the two regions.

**Figure 2:**
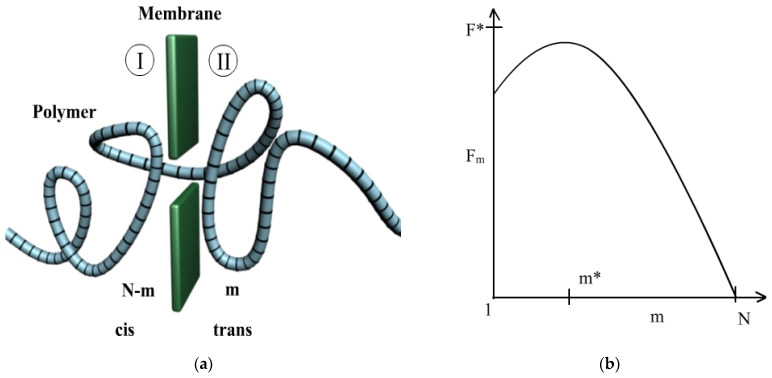
(**a**) Polymer escape in transition, (**b**) Associated free energy barrier (*m* segments in region II). m* is the value for which *F*(m*) = F*, respectively Fmaxim, meaning the maximum value of the function *F*, associated free energy barrier.

Figure 2 presents the polymer escape in transition, and the associated free energy barrier.

If γ is assumed to be identical on either side of the wall (when $\gamma_{1}$ is equal to $\gamma_{2}$)

(5) F(m)=(1−γ)k Tlnm(N−m)+mΔμ

In conclusion, the free energy of polymer translocation is a function of *m*. The second term in Equation (5), mΔμ, introduces a linear shift in the barrier’s baseline.

#### 2.1.2. Polymer Translocation through Pores

The polymer translocation through a nanopore under external fields has attracted considerable experimental and theoretical interest in recent years [3,4].

Following the usual scientific arguments of the classical transport theory, the motion of polymer in a potential field *U(x)* is described by the general equation [10,11,12]

(6) $$\frac{\partial G(x,t)}{\partial t}=-\frac{\partial J(x,t)}{\partial x}.$$

The G(x,t) represents the probability that the end segment coordinate of the polymer belongs to the interval [*x, x + dx*] and the flux probability J(x,t), is defined as

(7) $$J(x,t)=-\left[D(x)\frac{\partial J(x,t)}{\partial x}+\frac{1}{C(x)}\frac{\partial U(x)}{\partial x}G(x,t)\right].$$

The coefficient

(8) $$C(x)=\frac{1}{\beta D(x)}$$

is a total friction coefficient and $\beta^{-1}=k\;T$, where k = Boltzmann constant, respectively *T* = absolute temperature.

In Equation (6) the Einstein relation can be used, to connect the coefficient of diffusion by the frictional factor.

Frequently, the equation obtained from Equations (6) and (7)

(9) $$\frac{\partial G(x,t)}{\partial t}=\frac{\partial }{\partial x}\left[D(x)\frac{\partial G(x,t)}{\partial x}+\frac{1}{C(x)}\frac{\partial U(x)}{\partial x}G(x,t)\right]$$

is called the diffusion (Smoluchowski) equation [9].

If the potential function is the free energy *F*(*m*), (a function of *m* identical parts, or monomers, which have passed in trans zone) and *a* is the monomer dimension constant, where the likelihood that the final segment to belong in mathematical interval [*x*, *x* + *dx*] is different to zero, this function can be connected with the function of repartition, (a function of *m* and *t* variables) noted *P*(*m*,*t*), through a relation

(10) $$G(x,t)dx=\frac{1}{a}P(m,t)dm$$

then the Equation (7) becomes

(11) $$J(m,t)=-\frac{1}{a^{2}}\left[D(m)\frac{\partial P(m,t)}{\partial m}+\frac{1}{C(m)}P(m,t)\frac{\partial F(m)}{\partial m}\right].$$

Also, the Equation (6) in the new variables

(12) $$\frac{\partial J(m,t)}{\partial m}=-\frac{\partial P(m,t)}{\partial t}.$$

is a general differential equality of the diffusional dynamic into potential field, to a poor/reduced Reynolds number, [7].

Replacing into Equation (4), *D(x) = D_0_ = ct* and

(13) $$\frac{\partial U(x)}{\partial x}=U^{\prime }(x)-F$$

where *F* is the driving force and $U^{\prime }(x)$ is the friction force, we obtained a 1-D drift-diffusion model for the polymer translation through a nanopore.

(14) $$\frac{\partial G(x,t)}{\partial t}=D_{0}\frac{\partial }{\partial x}\left[\frac{\partial G(x,t)}{\partial x}+\frac{U^{\prime }(z)-F}{k_{B}T}G(x,t)\right].$$

In the equation above *G*(*x*,*t*) is a measure of the probability that a length *x* of the polymer backbone has passed the midpoint of the pore at the time *t* [3].

An intuitive model has been developed by M. Muthukumar [4,5,6]. Following the usual arguments of the classical nucleation theory (kinetics of nucleation) [13], the displacement of the polymeric suite via the nanomembrane is depicted by the

(15) $$\frac{\partial }{\partial t}P(m,t)=\frac{\partial }{\partial m}\left[k_{0}\frac{\partial }{\partial m}P(m,t)+\frac{k_{0}}{k_{B}T}\frac{\partial F(m)}{\partial m}P(m,t)\right]$$

where *P*(*m*,*t*) is the likelihood of detecting a nucleus of *m* segments in trans zone and $k_{0}$, independent of *N*, is the rate constant associated with the local friction in the transfer of one monomer through the hole.

In fact, the Equation (15) can be directly obtained from the Equations (11) and (12), considering $D(m)=k_{0}$, a nonuniversal constant.

As it can be observed, Equations (6)–(15) embed the diffusion equation as a basis, although the way of approaching the phenomenon is sometimes, different.

#### 2.1.3. Flux of Polymers

In this paper, we present in detail a one-dimensional diffusion model for describing the polymer motion through a nanopore. The interaction of the macromolecule with the nanopore will be taken into account by assuming that the diffusion occurs in the potential *U* and the diffusion coefficient depends on the particle position.

The behaviour of the polymers is described by *G*(*x*,*t*), the measure of the probability that a length *x* of the polymer backbone has passed the midpoint of the pore at a time *t*. The *G*(*x*,*t*) function satisfies the diffusion equation

(16) $$\frac{\partial }{\partial t}G(x,t)=\frac{\partial }{\partial x}\left\{D(x)e^{-\beta U(x)}\frac{\partial }{\partial x}\left[G(x,t)e^{\beta U(x)}\right]\right\}$$

identical with Equation (9).

There exist, now, two methods to solve these differential equations previously presented. In the first case, we are interested in the fact that we have a “channel” of finite length *L* that means that the polymer is still in the channel although the last monomer has passed the midpoint of the pore. In the second case, it is considered that the polymer passed through the pore if the last monomer passed the midpoint of the pore. This can also be translated into the initial and boundary conditions, in which the differential equation should be solved.

For the first case, *G*(*x*,*t*) is the density of the probability that the polymer can be found in the channel and then the initial condition is $G(x,0)=\frac{1}{L}$. The boundary conditions, imposed at the channel ends, describe the escape of the polymer from the channel. In general, to describe this, we use the radiation boundary conditions.

The second case will be exemplified in the equation

(17) $$\frac{\partial }{\partial t}P(m,t)=-\frac{1}{a^{2}}\frac{\partial }{\partial m}\left\{D(m)e^{-\beta F(m)}\frac{\partial }{\partial m}\left[P(m,t)e^{\beta F(m)}\right]\right\}$$

obtained from Equation (16), by imposing the relation (10), with *m*, a rightly ascertained variable with sluggish action on the full interval *0* ≤ *m < N*. Because *l* is the polymer length, *N =* $\frac{l}{a}$ is the number of polymer segments as before. The boundary conditions for the Equation (17) include acquaintance concerning the polymer external to the nanopore.

The entire state of absorption for the final segment (the last monomer of the polymer) is in the situation

(18) P(m=N,t)=0

because it is supposed that the complete passage of the polymer is irreversible; i.e., once the last monomer has passed through the nanopore, it will never again return.

Taking into consideration the relation (12), we can now write the Equation (17) in the form

(19) $$\frac{a^{2}}{D(m)}J(m,t)e^{\beta F(m)}=-\frac{\partial }{\partial m}\left\{P(m,t)e^{\beta F(m)}\right\},$$

which, using the relation (8) and by integrating in both sides [10,11], leads to

(20) $$a^{2}\beta \int_{0}^{N}J(m,t)C(m)e^{\beta F(m)}dm=-\int_{0}^{N}\frac{\partial }{\partial m}\left\{P(m,t)e^{\beta F(m)}\right\}dm.$$

Under the assumption of a stationary state (*J =* constant), in the boundary condition (Equation (18)), will be achieved

(21) $$J=\frac{P(m=0,J)}{a^{2}\beta \int_{0}^{N}C(m)e^{\beta [F(m)-F(0)]}dm},$$

an identical expression with Equation (7) from [1] and Equation (16) from [14].

The coefficient *C = C(m)* is a total friction coefficient, *a* is the lattice parameter and $\beta ={\left(k\;T\right)}^{-1}$, where k = Boltzmann constant and *T* = absolute temperature.

In a general case, the likelihood *P(m = 0,J)* that the last polymeric part (the last monomer) is at the nanopore admission aperture (nanopore inlet) is dependent on *J* [1].

#### 2.1.4. Results and Discussion I

A free energy expression *F*(*m*) of a polymeric suite with *m* parts (*m* monomers) in a semi-infinite face of penetration plane with a strong impenetrable surface at the origin and one end of the polymer constantly anchored at the heart of the barrier is obtained.

The general expression for the polymers flux through a small hole, under the anticipated circumstances, was derived. Nevertheless, the formula can be used if *C(m)* and *F*(*m*) are known.

Even though the results previously stated are useful in the quantitative knowledge of the polymer transport through a nanopore, the declared purpose of this work is to “produce” an explicit expression of the polymers flux.

It can be easily observed that the flux determination depends on the evaluation of the denominator of Equation (21)

(22) $$f(N)=a^{2}\beta \int_{0}^{N}C(m)e^{\beta [F(m)-F(0)]}dm.$$

The integral from the above equation may then be determined by numerical methods to achieve the expected concrete results.

Further on, we will discuss the undefined integral

(23) $$I(m)=\int C(m)e^{\beta [F(m)-F(0)]}dm.$$

and we will propose its solving in the case of some assumptions based on justified observations. There are two decisions that ease this computation, and the integral may then be analytically evaluated.

Firstly, we will explicitly write the dependency of the friction coefficient on *m,* respectively C=C(m). We can assume a linear dependence of the friction coefficient on the number of segments,

(24) $$C(m)=c_{1}m+c_{2}.$$

where *c_1_* is the frictional coefficient per pieces (monomer) in the nanopore (more precisely physical reciprocation between monomer and the membrane nanopore).

Secondly, we will explicitly write the dependence of the free energy on *m*, respectively F=F(m). The curve F=F(m), from Equation (5), graphically represented in Figure 2b, can be assimilated with a parabola, such that the assumption

(25) $$F(m)=-b_{1}m^{2}+b_{2}m+b_{3},b_{1}>0,$$

appears to be realistic and the difference of the free energies becomes

(26) $$F(m)-F(0)=-b_{1}m^{2}+b_{2}m,$$

and allows the computation of the integral

(27) $$I(m)=\int (c_{1}m+c_{2})e^{\beta (-b_{1}m^{2}+b_{2}m)}dm.$$

The result obtained by integration is

(28) $$\begin{array}{l} I(m)=\frac{1}{4\beta b_{1}{}^{3/2}}\{\exp[-\beta b_{1}m^{2}][\sqrt{\beta }(2c_{2}b_{1}+c_{1}b_{2})\sqrt{\pi }\exp[\frac{\beta b_{2}^{2}}{4b_{1}}+\beta b_{1}m^{2}]\\ \;\;\;\times erf(-\frac{\sqrt{\beta }(b_{2}-2b_{1}m)}{2\sqrt{b_{1}}})-2c_{1}\sqrt{b_{1}}\exp[\beta b_{2}m]]\}. \end{array}$$

A simple calculus leads us to

(29) $$\begin{array}{l} f(N)=a^{2}\beta [I(N)-I(0)]=\frac{l^{2}}{4N^{2}b_{1}{}^{3/2}}\{\sqrt{\beta }(2c_{2}b_{1}+c_{1}b_{2})\sqrt{\pi }\exp[\frac{\beta b_{2}^{2}}{4b_{1}}]\\ \times [erf(-\frac{\sqrt{\beta }(b_{2}-2b_{1}N)}{2\sqrt{b_{1}}})+erf(\frac{\sqrt{\beta }(b_{2}}{2\sqrt{b_{1}}})]+2c_{1}\sqrt{b_{1}}(1-\exp[\beta (-b_{1}N^{2}+b_{2}N)])\}. \end{array}$$

By the direct introduction of the *f(N)* expression from Equation (29) into the Equation (21), we obtain

(30) $$J=P(m=0,J){\left[f(N)\right]}^{-1},$$

the polymers flux through a small hole of nanometric size (“nanopore”).

In the case when the probability *P(m = 0,J)* is independent on *J*, we have the following equation

(31) $$J=P(m=0){\left[f(N)\right]}^{-1}.$$

Worth considering is the extensive work that deals with the transport process modelisation of polymers, having electrically charged monomers in composition, in the forces field with electrodynamic character [15]. In that review [15], the modeling of polymer translocation in the two distinct length regimes is presented, respectively short polymers and long polymers in the situation where these two effects are disconnected.

Thus, in the case of short/small polymers where polymer oscillations/fluctuations are insignificant, a rigid macromolecule model including the particularities of the electrohydrodynamic forces applied on the transporting molecule has been presented. We have thus described the dynamics of the polymer in a force field suitable for the process, which determines the translocation progress through porous membranes [15].

Note. The key parameter to describe the structural probability *P*(*m*,*t*) of a polymer chain is number of monomers *m* of the polymer situated in trans area. Basically, *P*(*m*,*t*) is the likelihood of detecting a nucleus of *m* segments in trans zone, independent of *N*.

An important theoretical description, performed recently, deals with the study of polymer translocation with monomers (or constituent segments) having opposite charges. The polymer chain moves under the mechanical effect of the superimposed external electric field, but in the presence of a pH gradient (the acidity or basicity specificity scale), by virtue of the Langevin dynamics equations. Everything is based on experimental observations, related to the electrostatic interaction between the membrane pores and the charged monomers of the macromolecule, which are influenced by the pH gradient modification [16].

Polymer translocation classical experiments are achieved by anionic polyelectrolytes such as DNA molecules (two threads that wind around one another, to realize a double helix/spiral) which move via negatively loaded membrane nanopores.

The whole philosophy stated above leads us to a system of differential equations of electrodynamics applied to an average level of the electric field, known as Smolukowski’s mathematical formalism [17].

### 2.2. Linear Polymers Transport under Constant Force

**Figure 3:**
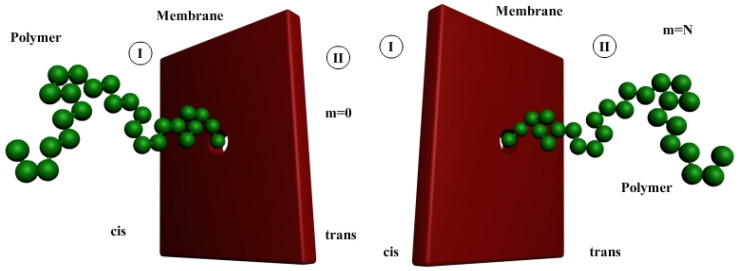
Polymer is found in *cis* zone (**left side**) and trans zone (**right side**).

In this section the linear polymers transport through a nanometer sized pore under the action of a constant force is presented. On this occasion the major mechanical effects of superimposed constant force and the monomers number of macromolecule on the polymer translocation process by nanomembranes have been demonstrated, in a 2D diffusion model [18,19]. A newer sequel, into a three-dimensional molecular dynamic, based on an external force, called “pulling force”, to stimulate translocation, can be read in the papers [20,21]. Brownian dynamics simulation is the most used molecular method for the polymers transport processes simulation, which postulates that the displacement mechanism of particles performing a Brownian motion is described in particular by Langevin equation. It must be said that it is almost impossible to solve the equations of motion analytically, so the only reasonable method is to numerically simulate. The general dynamics of each monomer results from the random bombardment of solvent molecules. In this conception, the monomer motion is evidently a Brownian motion. According to the announced scenario, any polymer molecule includes *N* monomers, each of dimension *a*, and is virtually forced to move from a cis zone to a trans area through a pore of nanometer size. Figure 3 depicts the three-dimensional representation of the two stages (initial and final) of the polymer location with respect to the membrane.

In a correct vision of the phenomenon studied, the movements of particles which follow Brownian motion dynamics are described by Langevin equation.

A classical formulation of Langevin differential equation, for some scalar γ≥0, which should meet the requirements advanced above, is the following expression

(32) $$\{\begin{array}{c} dx=vdt\\ dv=M^{-1}F\left(x\right)dt-\gamma vdt+\sqrt{2\gamma k\;T}M^{-1/2}dW(t) \end{array},$$

where *x* is the displacement vector, *v* is the velocity vector, *t* is the time, *M* is a diagonal matrix of masses, *F(x)* is the collective force vector, k is the Boltzmann constant, *T* is absolute temperature and *W(t), t > 0,* is a collection of independent standard Wiener processes. The above equation is written in compliance with the following conditions $k\;TD^{-1}$ = γM, where *D* is a constant diagonal diffusion tensor, *γ*(s^−1^) is dimensional scalar and *M* (Kg) is the diagonal matrix of masses, respectively. From a dimensional point of view, the product among γM represents the friction general tensor.

Regarding the standard Wiener process, we remember that *W*(0) = 0 with probability 1, respectively, when *Δt* > 0, the [*W*(*t* + *Δt*) − *W*(*t*)] difference is independent of *W*(*τ*) for *τ* < *t* and is Gaussian with mean zero and variance *Δt*.

Considering *v* as an auxiliary variable, the numerical methods have two components. The velocity definition can be arbitrarily chosen, and, most importantly, the position recurrence relation will determine the trajectory. For the Langevin equation, a Riemann-Stieltjes integral is adequate (and an Ito or Stratonovich interpretation is unnecessary).

Among the few possible variants of integration, it seems that the simple numerical Langevin integrator of the equation seems to be more appropriate for our conception.

The method further presented leads to an integrator’s form that uses positions only. This form is expressed in terms of values *x*, at which the force *F(x)* is evaluated and ensures the good accuracy of a numerical integrator. Direct integration, obviously made by parts, gives us

(33) $$\begin{array}{l} x(t)=x(t^{n})+\frac{1-e^{-\gamma (t-t^{n})}}{\gamma }v(t^{n})\\ +\int_{t_{n}}^{t}\frac{1-e^{-\gamma (t-s)}}{\gamma }M^{-1}F(x(s))ds\\ +{\left(2\gamma k_{B}T\right)}^{1/2}M^{-1/2}\int_{t_{n}}^{t}\frac{1-e^{-\gamma (t-s)}}{\gamma }dW(s). \end{array}$$

In the case of a constant force, *F(x(t)) = f = const.,* Equation (33) becomes

(34) $$\begin{array}{l} x(t)=x(t^{n})+\frac{1-e^{-\gamma (t-t^{n})}}{\gamma }v(t^{n})\\ +M^{-1}f\int_{t_{n}}^{t}\frac{1-e^{-\gamma (t-s)}}{\gamma }ds\\ +{\left(2\gamma k_{B}T\right)}^{1/2}M^{-1/2}\int_{t_{n}}^{t}\frac{1-e^{-\gamma (t-s)}}{\gamma }dW(s). \end{array}$$

The iterative calculus can be simplified without considering the thermal noise by neglecting the third term of Equation (34):

(35) $$\begin{array}{l} x(t)\approx x(t^{n})+\frac{1-e^{-\gamma (t-t^{n})}}{\gamma }v(t^{n})\\ +M^{-1}f\frac{e^{-\gamma (t-t^{n})}-1+\gamma (t-t^{n})}{\gamma^{2}} \end{array}$$

These results, respectively those illustrated by the expressions (33–35), were published for the first time by one of the authors in the paper [22].

#### Results and Discussion II

The Langevin equation can be solved by employing several known procedures, such as the time integration Ermak and Buckholtz [23] method, the van Gunsteren–Berendsen (vGB82) [24] and the Brooks–Brunger–Karplus (BBK) [25] algorithms, or the “Langevin impulse” (LI) integrator [22,25], for the case of a diagonal diffusion tensor. More to this, the interaction effect between immobile nanoparticle and polymer found in movement, on the membrane translocation process, was investigated using three-dimensional Langevin molecular dynamics [23].

We have studied the passage process of a translocating polymer of length *N* monomers, while it is pulled through a narrow pore with a constant force *F* applied to one end of the polymer. At small to moderate forces, satisfying the condition *FN^ν^*/*k*_B_*T* ≲ 1, where *ν* ≈ 0.588 is the Flory exponent for the polymer, we find that *τN*, the mean time the polymer takes to leave the pore, scales as *N*^2+*ν*^ independent of *F*, in agreement with our earlier result for *F* = 0. At strong forces, i.e., for *FN^ν^*/*k_B_T* ≫ 1, the behavior of the passage time crosses over to *τN*∼*N*^2^/*F*. We show here that these behaviors stem from the polymer dynamics at the immediate vicinity of the pore, in particular the memory effects in the polymer chain tension imbalance across the pore [26,27].

On the other hand, the electrical impedance of the pore increases with the entrance of a molecule as it displaces its own volume of the electrolyte solution.

By applying a voltage over the pore, the passing molecules are detected as current dips. For nanometer-sized pores (slightly larger than the molecule’s cross section), the magnitude and the duration of these dips have proved to be effective in determining the size and length of the molecules.

What is important is the action of the environment on the polymer engaged in the piercing movement of the membrane through the nanometer-sized pore. It thus suffers the influence of several forces, however different in nature, of which we shall discuss only two of them, known as additional forces. These supplementary forces which expressly act on the monomers, the viscous friction force $F_{r}=6\eta \pi av$(Stokes’ law) and the external electric force $F_{e}=qE$, due to the electric field activity (Coulomb’s law), have not been forgotten, whereas they are largely responsible for the realized transport process in a quasi-Newtonian viscous fluid (stationary flow characterizing), having a viscosity marked with η. The frictional force $F_{r}$, also known as drag force, is force manifested on a sphere of radius a (with which our monomer is approximated), moving through a fluid of viscosity *η* at speed *v*. By equalizing the two forces, it results that the steady gliding monomer velocity (in fact, the speed of all monomers, respectively the speed of the polymer as a whole) is roughly $v=\frac{qE}{6\eta \pi a}$.

**Figure 4:**
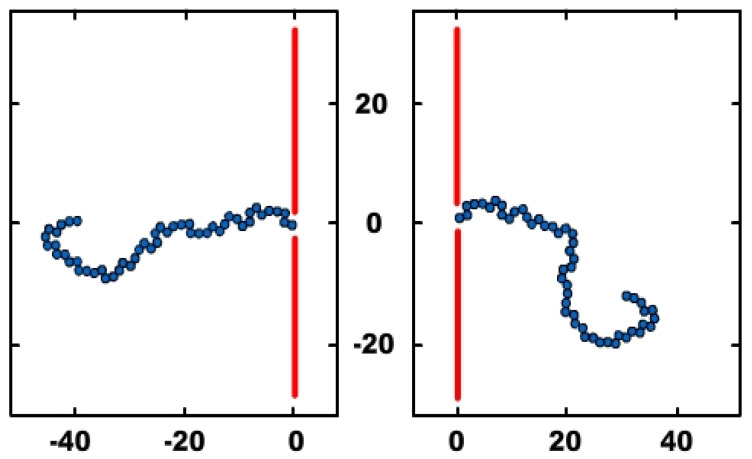
The polymer configurations of numerical computation, for *N* = 50. Initial position: polymer in *cis* zone (**left side**); final position: polymer in *trans* zone (**right side**).

Taking into account the expression of the electric force, equal to the product between the electric charge *q* and the intensity of the electric field *E*, we can refer with the same accuracy either to the effect of the force or to the effect of the electric field, when we speak of the influence of one of them. In Figure 4, the numerical computation configurations of *N* = 50 monomers in polymer, in the two hypostases, at the entrance to the nanopore of the membrane, respectively at the exit from it, are presented.

**Figure 5:**
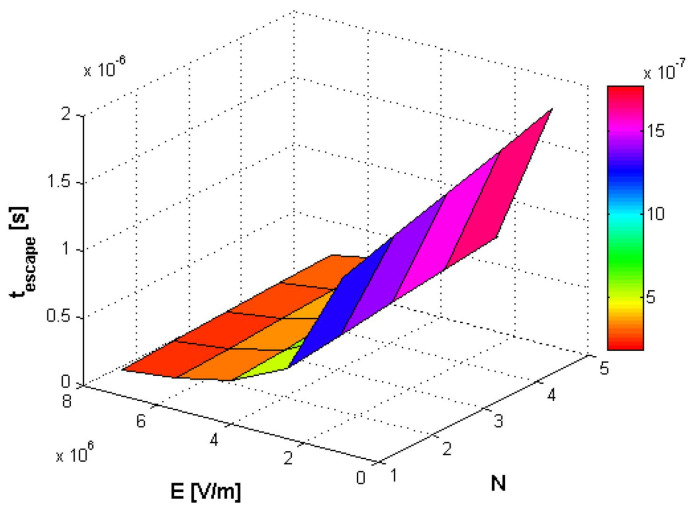
Simultaneous influence of both external electric field E (in the range [10^6^, 8 ∗ 10^6^]) and monomers number *N* of polymer (in the range [1, 5]), on the translocation time.

**Figure 6:**
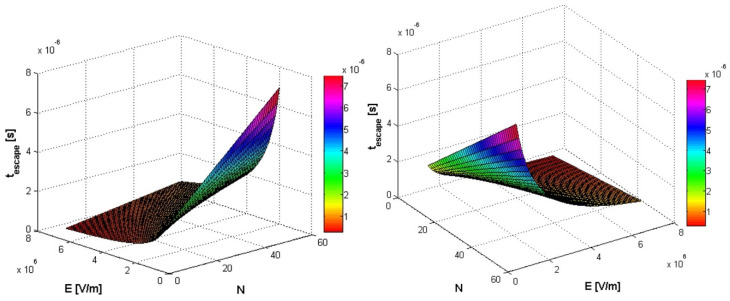
Simultaneous influence of both external electric field E (in the range [10^6^, 7 ∗ 10^6^]) and monomers number *N* of polymer (in the range [5, 50]), on the translocation time. Two different angles of presentation.

**Figure 7:**
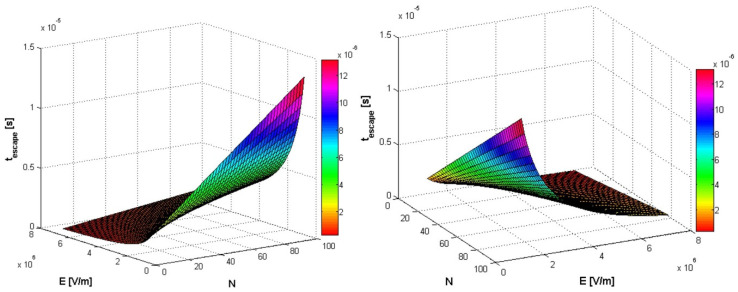
Simultaneous influence of both external electric field E (in the range [10^6^, 7 ∗ 10^6^]) and monomers number *N* of polymer (in the range [5, 100]), on the translocation time. Two different angles of presentation.

In Figure 5, Figure 6 and Figure 7, simultaneous influences of both external electric field E (in the range [10^6^, 7 ∗ 10^6^], in V/m units) and monomers number *N* of polymer (in the range [5, 50], [1, 5] and [5, 100]), on the translocation time in a three-dimensional graphic representation, are evaluated.

The model proposed, which aims to accurately describe the polymer transport trough nanometric sized pores, relies on a 2D drift-diffusion process and its associated equations [19]. The biopolymers translocation by the pores of membranes is found everywhere in biological systems, such as DNA and RNA (nucleic acids) transport beyond nuclear pores, or protein translocation across membrane channels, and virus inculcation to living cells. In the reference work [28], Meller et al. have thoroughly studied the blockade signals for the certain nucleic acids such as hetero-DNAs poly(dA50dC50) and poly(dAdC)_50_, because an undesirable DNA constituent inhabit in the nanopore blocks the current channel. The nanopore escape incidents are arranged in a pair of ably-localized categories with distinct blockage currents (flux intensity, undoubtedly), whose origin is still unclear, inexplicable. Another immediate parallel with the above study is detailed by the certitude that poly(dA) molecules show a top propensity to constitute a single-stranded base-stacked helices in comparison with poly(dC) [29]. The translocation process for two different nucleic acids of the hetero-DNAs poly(dAndCn) type has also been studied. The translocation time was experimentally measured (escape time, analogy) via nanomembrane in a complex investigation, and its histograms were drawn [30].

**Figure 8:**
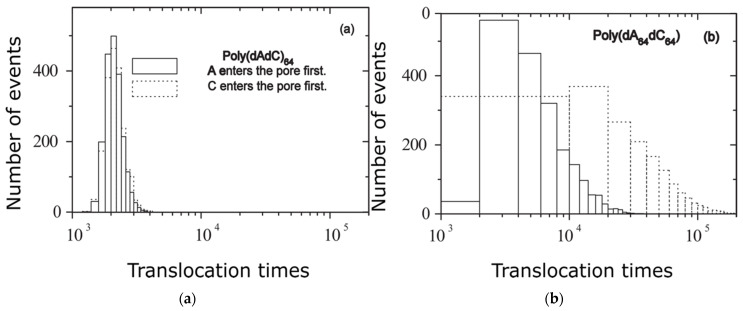
Translocation times histogram: (**a**) poly(dAdC)_64_ and (**b**) poly(dA_64_dC_64_) under *F* = 0.5.

In the case of block short length *M* = 2*n*, which appears in histograms of translocation time of Figure 8a, these histograms are conditioned poorly on the mechanical orientation, and the comportment is almost that of poly(dC).

Nevertheless, for a lengthy/large block dimension, the histogram strays/diverges noticeably from a Gaussian repartition having an oblong exponential queue, as can be easily seen in Figure 8b.

## 3. Conclusions

This paper confirms the understanding of recent experiments on the charged polymer membrane translocation and transport process of linear polymer under constant force. The driven translocation dynamics of a polymer chain through a nanopore is studied using a 2-D drift-diffusion model. The motion of polymer in a potential field is described by the Einstein–Smoluchowski general equation. The small dimension of the pore allows us to combine the dynamics of the pore’s interior and exterior. Outside the pore, the probability to find a final part (monomer) at the membrane aperture (in front of nanopore) is the critical parameter, which determines the polymers flux, *J*. Within the movement through nanopores, we can surmise that the polymer dynamics is depicted by the number of *m* parts (monomers), which have crossed the membrane aperture entrance (respectively nanopore).

In this context, the important theoretical description effectuated, deals with the study of polymer translocation with monomers (or constituent segments) having opposite charges. The polymer chain moves under the mechanical effect of the superimposed external electric field, but in the presence of a pH gradient (the acidity or basicity specificity scale), by virtue of the Langevin dynamics equations. Everything is based on experimental observations, related to the electrostatic interaction between the membrane pores and the charged monomers of the macromolecule, which are influenced by the pH gradient modification. The entire philosophy stated above leads us to a system of differential equations of electrodynamics applied to an average level of the electric field, known as Smolukowski’s mathematical formalism.

The integral from the denominator of general Equation (21) has been computed, assuming a linear dependency of the friction coefficient on the number of segments *m* and a parabolic behavior for the free energy *F*(*m*), in the company of an electric potential inside the nanopore. A precise formula of the polymers flux via a small hole, under the anticipated circumstances, was derived.

In the second theoretical chapter, the linear polymers transport through a nanometer sized pore under the action of a constant force is presented. The important dynamical effects of superimposed constant force and the monomers number of macromolecule on the polymer translocation process by membranes with nanometer-sized pores, in a two-dimensional diffusion model, have been demonstrated.

The model proposed, which aims to accurately describe the polymer transport through nanometric sized pores, relies on a drift-diffusion process and its associated equations. The biopolymers translocation by the pores of membranes is found everywhere in biological systems, such as DNA and RNA (nucleic acids) transport beyond nuclear pores, or protein translocation across membrane channels, and virus inculcation to living cells.

In the case of block short length *M* = 2*n*, which appears in histograms of translocation time of Figure 8a, these histograms are conditioned only poorly on the mechanical orientation, and the comportment is almost to that of poly(dC). Nevertheless, for lengthy/large block dimensions, the histogram strays/diverges noticeably from a Gaussian repartition having an oblong exponential queue, as can be easily seen in Figure 8b.

In order to exemplify the behavior of the polymer in the transport process through a nanometric-sized pore membrane, a 3D (three-dimensional) graphical representation of the simultaneous influence of both external electric field *E* and monomers number of polymer, on the escape time (named translocation time), was performed.

## Data Availability

The data used to support the findings of this study cannot be accessed due to commercial confidentiality.
